# The Influence of Circadian Rhythms on DNA Damage Repair in Skin Photoaging

**DOI:** 10.3390/ijms252010926

**Published:** 2024-10-11

**Authors:** Zhi Su, Qianhua Hu, Xiang Li, Zirun Wang, Ying Xie

**Affiliations:** 1 Key Laboratory of Molecular Epidemiology of Hunan Province, School of Medicine, Hunan Normal University, Changsha 410081, China; 2 Key Laboratory of Model Animals and Stem Cell Biology in Hunan Province, School of Medicine, Hunan Normal University, Changsha 410081, China

**Keywords:** ultraviolet radiation, DNA damage repair, circadian rhythms, skin photoaging

## Abstract

Circadian rhythms, the internal timekeeping systems governing physiological processes, significantly influence skin health, particularly in response to ultraviolet radiation (UVR). Disruptions in circadian rhythms can exacerbate UVR-induced skin damage and increase the risk of skin aging and cancer. This review explores how circadian rhythms affect various aspects of skin physiology and pathology, with a special focus on DNA repair. Circadian regulation ensures optimal DNA repair following UVR-induced damage, reducing mutation accumulation, and enhancing genomic stability. The circadian control over cell proliferation and apoptosis further contributes to skin regeneration and response to UVR. Oxidative stress management is another critical area where circadian rhythms exert influence. Key circadian genes like brain and muscle ARNT-like 1 (*BMAL1*) and circadian locomotor output cycles kaput (*CLOCK*) modulate the activity of antioxidant enzymes and signaling pathways to protect cells from oxidative stress. Circadian rhythms also affect inflammatory and immune responses by modulating the inflammatory response and the activity of Langerhans cells and other immune cells in the skin. In summary, circadian rhythms form a complex defense network that manages UVR-induced damage through the precise regulation of DNA damage repair, cell proliferation, apoptosis, inflammatory response, oxidative stress, and hormonal signaling. Understanding these mechanisms provides insights into developing targeted skin protection and improving skin cancer prevention.

## 1. Introduction

Ultraviolet radiation (UVR) is categorized into three types: UVA (320–400 nm), UVB (280–320 nm), and UVC (100–280 nm) [1]. The majority of UVC and a significant portion of UVB radiation are absorbed by the ozone layer, resulting in approximately 95% of the UVR reaching the Earth’s surface being UVA and 5% UVB [2,3]. The increased intensity of UVR, due to environmental pollution and ozone layer depletion, has become a major concern in recent years.

Prolonged or excessive UVR exposure can cause various types of skin damage, including irregular pigmentation, dry and rough skin, collagen degradation, and photoaging [4,5,6,7]. Skin photoaging not only impacts cosmetic appearance but also substantially elevates the risk of skin cancer [8]. UVR causes DNA damage primarily by generating cyclobutane pyrimidine dimers (CPDs) and 6–4 photoproducts (6–4PPs). If this damage is not effectively repaired, it can lead to mutations in oncogenes and tumor suppressor genes, ultimately resulting in skin cancers, including basal cell carcinoma, squamous cell carcinoma, and melanoma [9]. For instance, patients with xeroderma pigmentosum (XP), who lack functional nucleotide excision repair (NER) proteins, exhibit severe sensitivity to UVR. This condition significantly increases their risk of developing basal cell carcinoma and squamous cell carcinoma, with the likelihood of melanoma being up to 1000 times higher [10,11,12]. Therefore, understanding the mechanisms and protective measures against UV-induced skin photoaging is crucial for public health.

In modern society, disruptions in circadian rhythms, caused by shift work, transmeridian travel, and irregular sleep patterns, have become prevalent [13]. The circadian rhythm, or biological clock, is an endogenous time-tracking system that regulates physiological and behavioral activities to align with the Earth’s 24-h light–dark cycle [14]. In 2007 and 2019, the International Agency for Research on Cancer (IARC) classified shift work that involves circadian disruption as a Group 2A carcinogen or a probable carcinogen to humans [15,16].

Circadian rhythms are closely linked to skin homeostasis and regulate the skin’s sensitivity to UVR damage and its repair capacity [17,18]. Under normal circadian rhythms, the skin effectively defends against UVR-induced damage, reduces DNA damage, and promotes repair of damaged cells [19]. However, when the biological clock is disrupted, the skin’s self-protection and repair mechanisms can be impaired, leading to the increased accumulation of UVR-induced DNA damage. This, in turn, accelerates skin photoaging and elevates the risk of skin cancer [20].

This review aims to elucidate the impact of circadian rhythms on skin photoaging from UVR exposure. By integrating current knowledge on how circadian rhythms influence key physiological processes—such as DNA damage repair, cell proliferation regulation, apoptosis, inflammatory response, oxidative stress, and hormonal signaling—we provide a comprehensive overview of their effects on skin function. By elucidating these mechanisms, we have underscored the significance of circadian integrity in the preservation of skin homeostasis and its implications for the acceleration of photoaging. Our objective is to offer a nuanced understanding of how disruptions in circadian rhythms can contribute to the skin aging process induced by UVR exposure, thereby laying the groundwork for novel strategies for photoprotection interventions.

## 2. Impact of Circadian Rhythms on Physiological Processes

Circadian rhythms represent an endogenous time-tracking system that coordinates physiological processes to the 24-h light–dark cycle of the Earth. This system is comprised of two main components: the central clock situated in the suprachiasmatic nucleus (SCN) of the brain and peripheral clocks dispersed across various tissues and organs [21]. The central clock in the SCN is reset daily by morning sunlight entering the eyes [22]. Subsequently, the SCN synchronizes peripheral tissues, including the heart, lungs, liver, and skin, through neuronal and hormonal signals to maintain alignment with the central clock [23,24].

The circadian clock operates through two primary transcription–translation feedback loops (TTFLs). These loops generate substantial rhythmicity at the level of downstream clock-controlled genes, ensuring the stability and periodicity of circadian rhythms [25]. The fundamental mechanism depends on two key helix–loop–helix (bHLH)/PAS transcription factors: brain and muscle ARNT-like 1 (BMAL1) and circadian locomotor output cycles kaput (CLOCK). The BMAL1–CLOCK heterodimer binds to E-box sequences (CACGTG or CACGTT) in the promoters of target genes, driving rhythmic transcription of numerous clock-controlled genes (CCGs) [26]. As period protein (PER) and cryptochrome (CRY) protein levels increase, they accumulate in the cytoplasm and enter the nucleus, where they bind to the BMAL1–CLOCK complex, inhibiting the transcriptional activity of *PER* and *CRY* genes and thus closing the feedback loop of TTFLs [27,28,29].

In addition to the core feedback loops, an auxiliary loop engages the transcription factors retinoic acid-related orphan receptor (ROR) and reverse-erb (REV-ERB), which regulate the expression of *BMAL1*. ROR and REV-ERB competitively bind to retinoic acid-related orphan receptor response elements (ROREs) located in the promoter region of the *BMAL1* gene, with REV-ERB acting to suppress and ROR facilitating *BMAL1* transcription. This secondary feedback mechanism also influences circadian rhythms by modulating the abundance of BMAL1 [30,31,32,33]. Furthermore, an additional autonomous feedback loop involves the differentially expressed in chondrocytes (DEC) proteins, which are encoded by the basic helix–loop–helix family members basic helix–loop–helix family member E40 (*BHLHE40*) and basic helix–loop–helix family member E41 (*BHLHE41*) [34,35]. The BMAL1–CLOCK heterodimer induces the transcription of *DEC1* and *DEC2* through binding to E-box elements. However, unlike PER and CRY proteins, which directly inhibit the transcriptional activity of BMAL1–CLOCK, DEC1 and DEC2 compete with BMAL1–CLOCK for binding to E-box sites, thus repressing the transcription of all genes containing E-box elements, including those encoding PER, CRY, and DEC1/2 themselves [36,37].

Circadian rhythms directly and indirectly influence all metabolic processes and signaling pathways. Disruptions in circadian rhythms, caused by environmental factors or mutations in clock genes, can have detrimental effects on multiple organs [38]. Approximately 10–40% of the mammalian genome exhibits a 24-h rhythmic pattern, particularly in organs with significant metabolic functions, such as the liver, adipose tissue, pancreas, and muscles [39]. In liver metabolism, circadian rhythms regulate glucose, bile acids, lipids, and cholesterol levels [40]. Disruptions in circadian function can lead to lipid metabolism disorders, resulting in excessive fat accumulation in the liver and the progression to fatty liver disease. Additionally, circadian dysfunction can impair liver repair and regeneration, accelerating fibrosis and increasing the risk of cirrhosis and liver cancer [41].

Adipose tissue is not only a major energy reservoir but also an active endocrine organ [42]. The circadian mechanisms in white adipose tissue (WAT) regulate the expression of genes involved in lipid synthesis and breakdown, finely tuning lipid metabolism according to the circadian cycle [43]. Brown adipose tissue (BAT), on the other hand, primarily functions in non-shivering thermogenesis to generate heat and maintain body temperature. The circadian clock in BAT regulates the expression of thermogenesis-related genes, increasing their activity during the night or in cold environments to meet the body’s heat requirements. Circadian rhythms also influence fatty acid uptake and oxidation, making BAT more active during specific periods to produce heat [44,45].

Moreover, circadian disruptions are associated with obesity, metabolic syndrome, and glucose dysregulation. Mice with *CLOCK* gene mutations exhibit significant alterations in feeding rhythms, leading to overeating, obesity, and metabolic syndrome [46]. Prospective cohort studies in humans have shown that long-term shift work is linked to weight gain and an increased risk of type 2 diabetes [47]. Additionally, the electrophysiological characteristics of the heart display circadian rhythms, with a higher incidence of premature ventricular contractions and ventricular tachycardia/fibrillation occurring during daytime activities [48].

## 3. Circadian Regulation of Various Skin Functions

The skin, a highly complex and extensive organ, plays a crucial role in defending against various external stressors, including solar UVR and temperature fluctuations [49]. Similar to other tissues and organs in the body, skin functions such as transepidermal water loss, hydration, pH regulation, temperature control, and barrier function exhibit circadian rhythms [50]. These rhythms help the skin adapt to environmental changes, promote cellular proliferation and regeneration, and protect against aging and carcinogenesis [51].

The skin’s endogenous biological clock operates somewhat independently of the central clock. It regulates a wide range of genes’ circadian expression patterns even in the absence of central signals [52,53,54]. In mouse skin samples, at least 1400 genes have been identified with pronounced circadian expression patterns [52]. Similarly, hundreds of rhythmically expressed genes have been found in the human epidermis [55]. In keratinocytes, the expression of circadian genes, such as *CLOCK*, *PER1*, *PER2*, *PER3*, *CRY1*, *CRY2*, *REV-ERBα*, and *BMAL1*, shows significant rhythmicity [56,57,58]. In healthy male oral mucosa and skin tissue, the mRNA levels of these genes peak at different times of the day [59,60]. The PER protein in the circadian rhythm pathway inhibits the expression of matrix metalloproteinase-1 (MMP-1) via the cAMP signaling pathway, thereby reducing collagen degradation and delaying skin laxity and wrinkle formation. *PER2* and *PER3* knockout leads to a two- to three-fold increase in MMP-1 transcription levels [61].

Keratinocytes are the predominant cell type in the epidermis, accounting for over 90% of all epidermal cells. Genes that regulate keratinocyte differentiation are predominantly expressed late at night and in the early morning, whereas genes involved in the proliferation and differentiation of basal layer cells peak in the evening and at night. This suggests that the skin barrier is strongest in the afternoon, when hydration levels are at their peak, and that barrier moisture is at its lowest in the early morning [17].

Apart from keratinocytes, the epidermis also contains several other important cell types, each with specific functions. Melanocytes, located in the basal layer, are responsible for the production of melanin, a pigment that absorbs ultraviolet radiation and protects the skin from damage [62]. The synthesis of melanin is regulated by BMAL1. Knockdown of *BMAL1* reduces *PER1* transcription, and silencing *PER1* subsequently induces the phosphorylation of the microphthalmia-associated transcription factor (MITF), a key regulator of melanin production, thereby stimulating melanin synthesis and melanocyte activity [63,64]. In melanocytes, there also exists a light-sensitive protein, opsin 4 (OPN4), which increases skin pigmentation and protecting cells from photodamage. The expression level of OPN4 is associated with circadian rhythms, with low levels of OPN4 expression positively correlating with high levels of BMAL1 [65]. Circadian rhythms regulate OPN4, influencing not only the synthesis of melanin but also determining, to some extent, the extent of DNA damage in melanocytes following UVR exposure. Moreover, Langerhans cells, a specialized type of dendritic cell, were also influenced by circadian rhythms. In mice, the migration of Langerhans cells into skin lymphatics peaks during the day and reaches a low point at night [66]. This pattern indicates that the migratory capacity and immune function of Langerhans cells are regulated by circadian rhythms, potentially impacting skin immune responses and related physiological processes. Circadian rhythms also significantly influence the proliferation of epidermal stem cells. In mouse epidermis, there are three to four times more stem cells in the S phase (synthesis phase) at night compared to during the day. This is critical since the S phase is particularly sensitive to UVB-induced DNA damage [67]. Thus, the circadian clock reduces the proportion of epidermal stem cells in the S phase during the day—when UVB radiation is at its peak—allowing for more efficient DNA repair and minimizing potential damage from ultraviolet exposure. Moreover, the circadian clock regulates the DNA repair mechanisms and responses of epidermal stem cells to UVB radiation. It also impacts the metabolic states of cells at different times, influencing reactive oxygen species (ROS) levels and providing a degree of protection against oxidative damage [68].

In addition to epidermal and dermal cells, sebum production in the skin is also regulated by circadian rhythms. Studies have shown that facial sebum secretion peaks around noon and then gradually decreases at night, likely due to fluctuations in hormone levels [49,69]. Furthermore, in vitro studies of human hair follicle cells have revealed circadian rhythmic expression of core clock genes (*BMAL1*, *CLOCK*, and *PER1*) and clock-controlled genes (*c-Myc*, *NR1D1*, and *CDKN1A*), which are involved in regulating hair follicle growth, regression, and quiescence [54,70]. Mice lacking *BMAL1* exhibit premature aging across multiple tissues, a reduced lifespan, and early indicators of skin aging, such as delayed hair regrowth [52], thinning of the skin fat layer [71], and significant wound healing defects [72,73].

## 4. Role of Circadian Rhythm in Modulating Cellular Responses to UVR Exposure in the Skin

**Figure 1:**
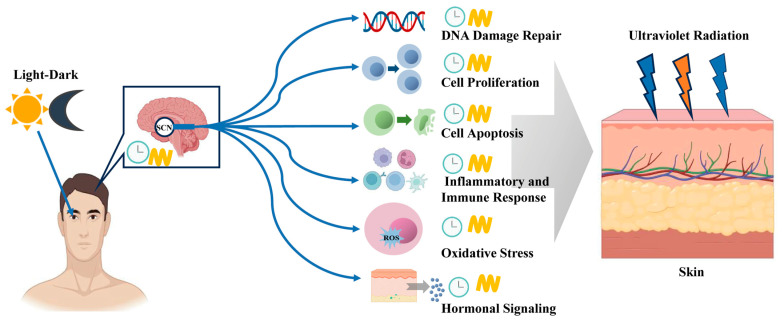
Key pathways in circadian modulation of UVR-induced skin damage.

As the outermost physical barrier of the body, the skin is daily exposed to UVR, which can lead to a variety of skin issues, including photoaging, sunburn, and the most lethal skin cancer—melanoma [74]. Human skin utilizes melanocytes in the epidermis to synthesize melanin and on the thickening of keratin in the stratum corneum to absorb and scatter UVR, thus protecting cells from damage [75]. When UVR penetrates the skin barrier and causes cellular damage, circadian expression and localization of various proteins are altered. For instance, narrow-band UVB radiation decreases *CRY2* expression in the epidermis and dermis but increases *CRY1* expression in subcutaneous fat [76]. PER1 expression decreases within 12 h of UVB exposure, while *BMAL1* and *CLOCK* expressions are suppressed at 20 and 24 h, respectively. Subsequently, these gene expressions recover and exhibit rhythmic variations [76,77]. UVC radiation affects the nuclear levels of the RNA-binding protein hnRNPR, which in turn impacts the post-transcriptional regulation of *CRY1* mRNA, and can induce the nuclear translocation of PER1 and CRY1 proteins post-UVC exposure [78]. As shown in Figure 1, these circadian changes impact mechanisms such as DNA damage repair, cell cycle control, apoptosis, and oxidative stress, profoundly influencing the process of UVR-induced skin photoaging [79,80].

### 4.1. DNA Damage Repair

The efficiency of DNA damage repair mechanisms fluctuates with circadian rhythms, with human DNA repair processes being more active during the day and less efficient at night [81]. Skin exposed to UVB radiation in the evening shows a higher erythema index (EI) compared to morning-exposed skin, and this circadian sensitivity is closely related to the expression level of *CRY2* in the skin [82]. The activity of DNA repair enzyme 8-oxoguanine DNA glycosylase 1 (OGG1) in human lymphocytes reaches its lowest at night and highest in the early morning, corresponding with changes in levels of 8-oxoG, a DNA damage marker [83]. Night shift workers exhibit lower baseline expressions of DNA repair genes, such as *ERCC1* and *OGG1*, compared to controls [84]. ERCC1 is involved in nucleotide excision repair, while OGG1 is responsible for base excision repair, and sleep deprivation further inhibits the expression of these genes, suggesting reduced DNA repair activity [84].

Disruption of circadian rhythms, such as those caused by shift work, significantly increases the risk of skin cancer, particularly melanoma [74]. Melanoma incidence is associated with the reduced expression of circadian genes such as *PER1, PER2, CLOCK, CRY1,* and *RORA*, which regulate critical cellular processes including the cell cycle, DNA repair, and apoptosis [85].

In contrast to nocturnal animals like mice, human skin’s circadian rhythm is aligned with diurnal activity. In mice, DNA repair efficiency is lower in the early morning [18], while activation of the BMAL1–CLOCK heterodimer and accumulation of *PER*/*CRY* mRNA occur during the day. Subsequently, PER–CRY proteins enter the nucleus at night, maintaining an endogenous rhythm of approximately 24 h [28,82,86]. Mice exposed to UVR in the early morning (when DNA repair activity is lower) show a higher susceptibility to skin cancer compared to those exposed in the evening (when DNA repair activity is higher) [87]. Loss of *BMAL1* results in the absence of circadian variation in DNA damage sensitivity, confirming BMAL1’s role in UVR-induced DNA damage responses [52].

Given that humans are diurnal, our skin’s DNA repair mechanisms are more efficient during the day and less so at night. This means that skin damaged by UVR at night has a reduced ability to repair, leading to increased damage accumulation. The loss of rhythmic expression in DNA repair genes, such as poly(ADP-ribose) polymerase 1 (*PARP1*) and *RAD50*, in shift workers heightens their sensitivity to DNA damage [88]. Reduced expression of breast cancer type 1 and type 2 susceptibility genes (*BRCA1* and *BRCA2*) also increases the risk of breast cancer [89]. Epidemiological evidence suggests that circadian disruption from shift work may increase skin cancer risk [90,91]. Consequently, the International Agency for Research on Cancer (IARC) has classified shift work as a potential carcinogen [15].

UVR primarily causes skin damage through DNA lesions, including CPDs, 6–4PPs, base oxidation products, and double-strand breaks. To address these extensive DNA damages, the body has developed complex and precise repair mechanisms to maintain genomic integrity and proper cellular function.

#### 4.1.1. Nucleotide Excision Repair (NER)

The NER is a critical pathway for addressing UVR-induced damage, such as CPDs and 6–4PPs [92,93,94]. NER is a multi-step process that identifies and removes damaged DNA segments, followed by DNA synthesis and sealing to repair the remaining gaps in the DNA backbone [93,95]. A key rate-limiting component of NER, the XPA protein, exhibits high affinity for pyrimidine dimers and binds to and marks the damaged DNA strand [96].

Circadian rhythm genes directly influence the efficiency of NER by dynamically regulating XPA protein levels [97]. *XPA* mRNA and protein levels show circadian rhythms in various tissues, including the mouse brain [98], liver [99], and skin [18]. During periods of high circadian gene activity, XPA expression levels are elevated, enhancing DNA repair efficiency. Conversely, when circadian genes are less active, XPA levels decrease, resulting in reduced repair efficiency [66]. In SKH-1 hairless mouse models, the clearance rate of UVR-induced damage is higher in the evening compared to the morning, correlating with increased XPA protein expression [18]. The BMAL1–CLOCK complex promotes XPA expression, whereas the CRY–PER complex suppresses it [18,98]. Time-restricted feeding can alter the phase and amplitude of the skin’s circadian clock, affecting the peak expression of XPA or altering its rhythmic expression. This change can make skin that is normally more sensitive to UVB at night more sensitive during the day [100]. Additionally, the combined use of REV-ERB antagonist SR8278 and CRY inhibitor KS15 in HaCaT cells results in a two- to three-fold increase in *XPA* mRNA levels and a 1.5-fold increase in protein expression [101].

In the final stage of NER, DNA ligase I (LIG1) is responsible for joining newly synthesized DNA fragments with the original DNA strand, thus processing Okazaki fragments and ensuring the effective replication of damaged DNA [102,103]. TIMELESS (TIM), by interacting with PARP1, facilitates the repair of ssDNA gaps associated with replication forks, thereby supporting the role of LIG1 in DNA ligation [104].

#### 4.1.2. Base Excision Repair (BER)

For smaller chemical modifications such as base oxidation damage, BER plays a crucial role [105]. BER involves specific glycosylases that recognize and remove damaged bases, followed by DNA polymerase β (POLβ) filling in the missing nucleotides, and finally, DNA ligase completing the repair [106]. X-ray repair cross-complementing protein 1 (*XRCC1*) is a key gene involved in BER. XRCC1 stabilizes the ends of DNA single-strand breaks and assists in the accurate positioning of DNA polymerase β to synthesize missing nucleotides, interacting with DNA ligase IIIα to promote rejoining of the DNA strand and restoring DNA continuity [107]. The expression and function of XRCC1 are influenced by the p53 protein, which is activated in response to UVR damage and enhances XRCC1 accumulation at damage sites, further improving repair efficiency [108]. Core circadian genes such as *CLOCK*, *BMAL1*, *PER*, and *CRY* can affect the expression patterns of DNA repair proteins, including XRCC1. For example, PARP1 enzyme activity is subject to cyclical regulation by BMAL1–CLOCK and PER–CRY complexes [109]. High expression phases of BMAL1–CLOCK are often associated with increased PARP1 activity, while high expression phases of PER–CRY may suppress PARP1 activity, ensuring that DNA repair occurs at optimal times [110]. PARP1 interacts with XRCC1 through ADP-ribosylation, affecting the assembly and stability of DNA repair complexes. During phases of high PARP1 expression or activity, XRCC1 recruitment and stability are typically increased. Additionally, XRCC1 promotes the localization and repair of damaged DNA by binding to POLβ. Interaction between PER1 and POLβ regulates the circadian expression of POLβ, and fluctuations in POLβ expression can impact XRCC1 recruitment and function, thereby affecting DNA repair efficiency [111].

#### 4.1.3. Translesion Synthesis (TLS)

In cases where damage is too severe for high-fidelity DNA polymerases to bypass, TLS serves as a tolerance mechanism. TLS employs low-fidelity polymerases, such as POL η and POL κ, to bypass or tolerate damaged sites, allowing the replication fork to continue and ensuring DNA replication continuity, albeit at the cost of fidelity [112]. REV1 acts as a scaffold protein in TLS, binding to DNA damage sites and recruiting DNA polymerase ζ (POL ζ) and other Y-family TLS polymerases, such as POL κ, POL ι, and POL η, through interactions with REV7 [113]. REV1 is involved in TLS and assists cells in completing replication in the presence of DNA damage [114], and its expression may be regulated by circadian rhythms [115].

#### 4.1.4. Double-Strand Break Repair (DSBR)

Severe DNA damage, such as double-strand breaks (DSBs), triggers the double-strand break repair mechanisms, primarily involving homologous recombination (HR) and non-homologous end joining (NHEJ) [116]. HR relies on a homologous chromosome as a template for precise repair, while NHEJ directly joins the broken ends, which may result in sequence loss or insertion [117].

It has revealed that CLOCK binds to the promoter regions of DNA damage repair genes such as *p21*, *NBR1*, *BRCA1*, and *RAD50*, suggesting its role in enhancing DNA repair capabilities against UVR-induced damage [118]. CLOCK’s interaction with RAD50 enhances cellular repair capacity for UVR-induced DNA damage [118]. Integrating transcriptomic and cistrome data has shown that CRY1 regulates the expression of key homologous recombination repair factors, including RAD51, BRCA1, BRCA2, XRCC3, and CHEK1, in a circadian manner by binding to their promoter regions [119]. Specifically, CRY1 binds to the promoters of DNA repair sensors like MRE11 and RAD50 early after DNA damage and later regulates the expression of downstream HR components, such as XRCC3 and POLD2 [119]. PER2 has also been observed to promote the expression of homologous recombination factors, including MRE11 and NBS1, thereby enhancing the function of the RAD50–MRE11–NBS1 (MRN) complex and improving DNA repair capacity in mouse fibroblast cells (NIH 3T3) [120]. In colorectal cancer patients, PER2 has been found to enhance the expression of homologous recombination repair factors, thereby boosting the DNA damage response [121].

BRCA proteins play a crucial role in homologous recombination repair by recruiting and stabilizing RAD51, which forms filaments on damaged DNA strands, a key step in the repair process [122]. Carriers of BRCA1 and BRCA2 mutations, who are unable to perform effective homologous recombination repair, face significantly increased risks of breast and ovarian cancers [123]. The circadian rhythm proteins PER1 and PER2 interact with BRCA1, which is crucial for the regulation of the homologous recombination (HR) repair pathway, affecting the ability to repair DSBs [124,125]. BRCA1, a well-known tumor suppressor protein, has been shown to interact with circadian proteins PER1 and PER2 through yeast two-hybrid experiments [124]. In estrogen receptor-negative breast cancer samples, decreased expression of *PER1* and *PER2* suggests that circadian genes may regulate homologous recombination repair capacity [124]. The mutations in *BRCA1* and *BRCA2* increase the risk of developing breast and ovarian cancers. REV-ERBα (NR1D1) in the auxiliary loop is recruited to DNA damage sites after PARylation modifications, inhibiting the recruitment of BRCA1, SIRT6, and pNBS1 to double-strand break sites [126].

Circadian gene expression may influence BRCA protein activity and stability, thereby affecting its role in DNA damage repair [125]. At the cellular level, BMAL1 binds to the promoter regions of BRCA1 and BRCA2, providing protective effects against DNA damage induced by ionizing radiation [127]. Additionally, CLOCK interacts with BRCA1 to prevent UVR-induced apoptosis, enhancing cellular tolerance to UVR [118]. CRY1 has also been found to promote *BRCA1* transcriptional activation, further enhancing cellular repair capacity by increasing BRCA1 levels [26].

UVR irradiation induces larger DNA lesions, such as pyrimidine dimers, which interfere with replication fork progression and activate the ATR pathway [128]. ATR kinase is recruited to damage sites by single-strand DNA-binding proteins (such as RPA) and ATRIP (ATR interacting protein), where it maintains fork stability and, through the phosphorylation of recombination proteins like RAD51 and BRCA2, helps form intermediate complexes required for homologous recombination [129]. Rhythmic circadian activity is essential for ATR function. CRY1 interacts with TIM to modulate ATR-mediated DNA damage checkpoint responses at specific times of the day, aiding in repair processes in mouse embryo NIH3T3 fibroblasts after UVR exposure [130]. Depletion of *CRY1* by small interfering RNA (siRNA) abolishes circadian-dependent ATR activity [130]. When ATR kinase loses its circadian-dependent regulation, it cannot effectively respond to UVR-induced DNA damage, leading to delayed or reduced repair efficiency, an increased sensitivity to UVR damage, and potentially accelerated apoptosis or DNA damage accumulation, which may increase cancer risk over time [131].

#### 4.1.5. Single-Strand Break Repair (SSBR)

The SSBR is a crucial mechanism for skin cells to address UVR-induced damage, specifically targeting single-strand breaks that occur either as a result of base excision repair or directly due to UVR radiation. A key protein in the SSBR pathway is X-ray repair cross-complementing protein 1 (XRCC1) [132]. While XRCC1 does not possess catalytic activity, it functions as an essential scaffold protein that coordinates and facilitates interactions between various repair enzymes, including DNA polymerase β (POL β), DNA ligase IIIα (Ligase IIIα), and Poly(ADP-ribose) polymerase 1 (PARP1) [133]. XRCC1’s role in assembling and stabilizing these components is vital for efficient DNA repair.

Together with other DNA repair mechanisms, SSBR constitutes a primary defense line for skin cells against UVR-induced damage, playing a crucial role in preventing photoaging and skin cancer. However, when the repair capacity is exceeded or efficiency diminishes, the accumulated DNA damage can lead to structural and functional degradation of the skin, increasing the risk of skin diseases. Thus, understanding how circadian rhythms influence these repair pathways and their regulatory mechanisms is critical for developing new strategies for preventing and treating UVR-induced skin damage.

There are certain proteins that play pivotal roles across multiple DNA damage repair pathways, acting as versatile guardians of genomic integrity. Poly (ADP-ribose) polymerase 1 (PARP1) is another pivotal protein involved in several DNA repair pathways [134]. PARP1 detects damaged sites within chromatin and regulates the activity of DNA repair proteins through ADP-ribosylation, initiating processes involved in nucleotide excision repair, base excision repair, single-strand break repair, and double-strand break repair [135,136,137]. PARP1 activity peaks during the active phase and diminishes during sleep. In mouse liver, PARP1 enzyme activity exhibits a circadian rhythm, with UVR-induced DNA repair mediated by PARP1 being more effective during sleep, aligning with the peak demand for cellular repair [138].

### 4.2. Regulation of Cell Proliferation

Frequent cell proliferation is essential for maintaining the health and function of skin tissue [139,140]. In human oral mucosa and skin biopsies, *PER1* mRNA expression peaks in the morning, aligning with the peak expression of p53, a marker for the G1 phase of the cell cycle. In contrast, *BMAL1* mRNA levels reach their highest in the evening, synchronizing with the peak expression of cyclin B1, a marker for the G2 and M phases of the cell cycle [59,141]. This indicates that DNA synthesis and cell proliferation in skin tissues also follow a 24-h rhythm [142]. Cells are most susceptible to DNA damage during the peak of DNA synthesis, which is a critical phase for UVR sensitivity [52]. Genes related to the cell cycle, such as *CDC20*, *CDC25B*, *KIF20A*, and *WEE1*, may regulate mitosis and facilitate DNA repair in epidermal cells at specific times of the day following UVR exposure [55].

In human skin tissue, UVR exposure is reduced at night, allowing cells to perform more efficient DNA repair, whereas during the day, especially when solar radiation is at its peak, cells accelerate proliferation with heightened DNA replication activity. Mice that are irradiated in the morning, when DNA repair activity is lower and DNA synthesis is higher, show a greater risk of developing skin cancer compared to mice irradiated in the evening, when DNA repair activity is higher and DNA synthesis is lower [52]. The sensitivity of mouse epidermal cells to UVR-induced DNA damage is time-dependent and relies on BMAL1. Keratinocytes exhibit a pronounced circadian rhythm in DNA replication, which is disrupted in *BMAL1*-deficient mice, resulting in a constant and elevated proportion of S-phase cells and indicating that BMAL1 plays a role in suppressing epidermal cell proliferation [52].

The circadian protein CRY1 also plays a crucial role in cell cycle regulation. CRY1 interacts with the TIM protein and is central to DNA replication. This interaction promotes the phosphorylation of mini-chromosome maintenance protein 2 (p-MCM2), a key regulator in DNA replication. Elevated levels of p-MCM2 contribute to the accuracy and integrity of DNA replication [130]. CRY1 enhances the mRNA levels of *CDKN1A* (cyclin-dependent kinase inhibitor 1A), which inhibits cyclin-dependent kinases (CDKs) at various cell cycle checkpoints, allowing cells to arrest at G1/S, G2/M, or S phase checkpoints and ensuring that cells divide only after DNA is fully replicated and any damage is repaired [143]. The depletion of *CRY1* significantly impairs this process, whereas CRY1-stable cells exhibit moderate upregulation of CDKN1A, further confirming CRY1’s role in cell cycle checkpoint control and cell proliferation following DNA damage [119]. In DNA damage models of cancer cells, CRY1 undergoes phosphorylation and deubiquitination, increasing its stability [144]. CRY1 also regulates the expression of cell cycle regulators such as Wee1 and p21, helping maintain G1-phase cells in repair and preventing premature progression to S phase [145].

CRY1’s interaction with TIM finely tunes the circadian activity of ATR kinase [130]. Once activated, ATR phosphorylates downstream effectors such as CHK1, which in turn inhibits the activity of CDC25 phosphatases, thereby blocking the transition from G2 to M phase of the cell cycle [146]. This regulatory mechanism provides cells with sufficient time to perform nucleotide excision repair (NER) and ensure timely DNA repair, preventing genomic instability and cancer risk. However, a study on mouse skin fibroblasts suggests that the downregulation of CRY1 may not directly affect ATR activation, indicating that the network of DNA damage responses involves multiple regulatory mechanisms, and CRY1’s role may be more subtle or dependent on specific cellular contexts and conditions [147].

KLF9, a transcription factor, regulates keratinocyte proliferation in human epidermis [148]. The BMAL1–CLOCK complex activates the E-box elements in the *KLF9* promoter region, modulating *KLF9*’s oscillatory expression and its anti-proliferative effects, finely tuning the rhythm of keratinocyte proliferation and differentiation to maintain normal skin physiology [148].

The p16 and p21 are critical cell cycle regulators that play key roles during the transition from G1 phase (pre-DNA synthesis) to S phase (DNA synthesis). p16 inhibits CDK4 and CDK6 by binding directly to them, preventing their interaction with cyclin D and keeping Rb protein in a non-phosphorylated state, thereby blocking E2F activation and entry into S phase [73]. The p21 binds to cyclin E/CDK2 complexes, inhibiting their activity and preventing cells from entering S phase, thus halting the initiation of DNA replication [149]. Non-POU-domain-containing octamer-binding protein (NONO) activates p16 transcription in a PER-dependent manner. Knockout of *PER* or *NONO* results in insufficient activation of p16, leading to reduced cellular senescence and increased proliferation [73]. Conversely, *BMAL1*-deficient cells show *p21* overexpression through reduced REV-ERB levels or increased RORγ levels, leading to decreased cell proliferation [149]. The classic tumor suppressor protein p53 activates *p21* transcription following DNA damage, pausing the cell cycle to allow DNA repair [150,151,152]. When PER2 binds to p53, it impairs p53’s transcriptional activation capacity, indirectly regulating cell cycle progression by preventing effective activation of downstream target genes such as *p21* [153].

Circadian rhythms also influence the transition from G2 to mitosis (G2/M) [154]. WEE1, a serine/threonine kinase, acts as a “brake” in the G2/M transition by phosphorylating and inhibiting CDK1, thus preventing premature entry into mitosis [155]. *WEE1* transcription is activated by the BMAL1–CLOCK complex and inhibited by PER/CRY proteins [156]. Cyclin B1, a key factor that forms an active complex with CDK1, acts as an “accelerator” during the G2/M transition by dephosphorylating and activating CDK1 to promote mitotic entry. Cyclin B1 synthesis increases at specific times of the day, forming more complexes with CDK1 and driving cell cycle progression. The BMAL1–CLOCK complex regulates Cyclin B1 expression to ensure proper cell cycle progression [157]. Depletion of BMAL1–CLOCK complex expression results in extended cell cycle duration, providing strong evidence that BMAL1–CLOCK may function more as an activator rather than a repressor in cell cycle regulation [158,159].

Cell cycle-related proteins also feedback to regulate circadian rhythms. CDK1 phosphorylates REV-ERBα, marking it for ubiquitination and degradation by FBXW7, thus increasing the CLOCK and BMAL1 expression [160]. This interaction between circadian rhythms and cell cycle regulation coordinates cell speed and efficiency.

### 4.3. Cell Apoptosis

Circadian rhythm genes play complex and multifaceted roles in regulating DNA damage and apoptosis in cancer cells. In NCI-H460 lung cancer cells and HCT-116 colon cancer cells, the expression of *PER1* and its subsequent suppression do not significantly affect ionizing radiation (IR)-induced apoptosis [161]. However, in other human cancer cell lines, such as CA9-22 pancreatic cancer cells, MTA PCa-2 prostate cancer cells, and HepG2 liver cancer cells, PER1 exhibits anti-apoptotic properties. Knockdown of *PER1* increases apoptosis induced by cisplatin [162,163]. Conversely, the downregulation of *PER1* interferes with the phosphorylation of Chk2 by ATM triggered by IR, reducing drug-induced apoptosis resulting from double-strand DNA breaks [92,164]. This suggests that the overexpression of *PER1* may enhance the sensitivity of human cancer cells to DNA damage-induced apoptosis, while its inhibition weakens the apoptotic response.

Circadian rhythm genes also play critical roles in regulating apoptosis in response to UVR exposure. Following UVB irradiation, the decreased expression of *BMAL1* and *CLOCK* genes inhibits the apoptotic response induced by UVB [165]. In mice with double knockout of *CRY1* and *CRY2 (CRY1/2^−/−^)* or *PER1* and *PER2 (PER1/2^−/−^)*, the time-of-day effects on sunburn-induced apoptosis are eliminated [166].

The tumor suppressor p53 responds to DNA damage signals by initiating repair mechanisms [167]. Mutations in the *p53* gene significantly increase the risk of skin lesions under UVR exposure [168,169]. In human skin, the baseline expression of CRY2 correlates with erythema response after UVR exposure [82]. CRY2 enhances the skin’s defense against UVR by regulating p53 protein levels, with higher p53 protein levels observed in morning-exposed subjects compared to those exposed in the evening [82,166]. In UVR-irradiated mouse embryonic fibroblast NIH3T3 cells, the interaction between heat shock factor 1 (HSF1) and BMAL1 enhances p53’s transcriptional activity. This coordination stabilizes the CRY1 protein and prevents apoptosis [170]. The use of mutp53 reactivators, such as SLMP53-2, to reactivate mutated p53 proteins, when applied topically to mouse skin, reduces UVB-induced apoptosis by inhibiting c-Jun N-terminal kinase (JNK) activity [9].

When damage cannot be repaired, p53 activation triggers apoptotic pathways to eliminate damaged cells. In the morning, under the control of the body’s circadian rhythm, p53 protein levels peak, making cells more prone to initiating p53-dependent apoptotic pathways [171]. Upon DNA damage or other stress, the expression levels of PER1 and PER2 rise. These proteins interact with p53, enhancing p53 stability and promoting p53-dependent apoptotic cascades [172]. By eliminating potential cancer cells, p53 provides a protective barrier for the skin. However, prolonged UVR exposure often impairs p53’s pro-apoptotic functions due to mutations [166]. PER2 protein, in collaboration with CRYs, regulates the expression of Mdm2 protein, which is at lower levels in the morning, reducing its interaction with p53 and thus decreasing p53 ubiquitination and degradation [173]. This results in increased accumulation of p53 protein in the morning and triggers a stronger p53-dependent apoptotic response following UVR exposure [166]. Ozturk et al. found that *p53^–/–^CRY1/2^–/–^* cells are more sensitive to genotoxic drugs compared to *p53^–/–^* cells, with *CRY* mutations making *p53*-mutant cells more prone to apoptosis under genotoxic stress [174]. In *p53*-mutant cells, CRY protein deficiency leads to excessive phosphorylation and inactivation of glycogen synthase kinase 3 beta (GSK3β), which impedes normal phosphorylation and activation of GSK3β’s anti-apoptotic activity, making cells more susceptible to apoptosis [175].

In addition to p53’s role in apoptosis regulation, p73—a protein that becomes a secondary clock-controlled gene after DNA damage—takes on crucial apoptotic regulatory functions in *p53*-mutant cells. Its expression and function are directly regulated by the circadian clock [176]. In the absence of DNA damage, p73 is typically expressed at low levels. After UVR exposure or DNA damage caused by UVR-mimicking agents like oxaliplatin, low expression of CRY proteins enhances the binding of early growth response protein 1 (EGR1) to the *p73* promoter, thereby activating p73 and promoting apoptosis [177]. At this time, EGR1 with E-box is considered a primary CCG, while p73 acts as a secondary CCG [177]. Additionally, CRY2 cooperates with the E3 substrate receptor FBXL3 to enhance c-MYC degradation, which helps reduce apoptosis induced by UVR exposure, thereby inhibiting skin cancer development [178]. Overall, the loss of CRY proteins affects not only the cellular clock mechanism but also disrupts several key apoptotic signaling pathways, altering skin cells’ sensitivity to apoptotic inducers following UVR exposure.

### 4.4. Inflammatory and Immune Response

Acute UVR exposure can trigger an inflammatory response in the skin, typically marked by vasodilation and heightened blood flow in the dermis, leading to erythema at the site of sunburn [179,180]. In SKH-1 hairless mice exposed to UVB in the morning, the severity of sunburn erythema is significantly greater compared to evening exposure, indicating that morning UVR exposure leads to increased apoptosis in sunburned skin cells and exacerbates the inflammatory response [181]. Similarly, human subjects exposed to UVA radiation in the morning often experience more pronounced erythema compared to those exposed in the evening. This difference is reflected in more significant vasodilation, a heightened inflammatory response, and increased apoptosis [181]. These findings underscore the critical role of circadian rhythms in regulating the skin’s inflammatory response to UVR exposure.

Core clock genes, *PER* and *BMAL1*, regulate the circadian expression pattern of tissue inhibitor of metalloproteinases 3 (TIMP3), ensuring its peak expression during the day to respond to and adapt to various environmental stresses, including UVR. Following UVB exposure, UVB can rapidly inhibit *CLOCK* gene expression, although this effect normalizes after 12 h [182]. CLOCK forms a transcriptional activation complex with BMAL1, positively regulating TIMP3 expression. Reduced CLOCK expression leads to the downregulation of TIMP3, which in turn increases the secretion of TIMP3-targeted molecules, such as integrin metalloproteinases (e.g., ADAM17) and pro-inflammatory cytokine tumor necrosis factor-alpha (TNF-α), thereby promoting the inflammatory response triggered by UVB [183]. Park et al. also observed decreased CLOCK and TIMP3 expression in UVB-irradiated human keratinocytes, leading to the derepression of inflammatory factors such as MMP-1, TNF-α, CXCL1, and IL-8, which contributes to the development and progression of skin inflammation [182]. Although MMP-1 is not traditionally classified as an inflammatory factor, its expression is usually upregulated during inflammation because it is involved in tissue remodeling at inflammatory sites, a key pathological feature of many inflammatory diseases. Exposure to UVR induces MMP-1 expression and DNA damage, with PER proteins, as key components of the circadian rhythm system, inhibiting MMP-1 expression in human keratinocytes [61].

REV-ERBα, a known nuclear receptor, suppresses NF-κB activity, indirectly controlling the expression of pro-inflammatory cytokine IL-6 in mouse macrophages. In experimental colitis mouse models, REV-ERBα demonstrates significant anti-inflammatory properties by specifically inhibiting NF-κB and Nlrp3 expression, effectively reducing Nlrp3 inflammasome activity [184]. These findings suggest that circadian fluctuations may influence REV-ERBα expression levels, thereby affecting the activity of inflammatory factors such as NF-κB and Nlrp3, ultimately modulating the degree of skin inflammation following UVR exposure.

UVR exposure triggers a series of immune responses [185]. High doses of UVB significantly reduce the number of Langerhans cells (LCs) in the epidermis. LCs are specialized antigen-presenting cells responsible for capturing and presenting antigens to T cells, initiating an immune response. A decrease in LCs can weaken the skin’s immune surveillance, reducing its ability to recognize and eliminate pathogens and abnormal cells [186]. In addition to the reduction in number, UVB can impair LCs’ functionality, including diminished antigen presentation capability and lymphocyte activity, decreased surface antigen expression, and lower levels of stimulatory molecules such as B7, directly impacting the initiation and strength of the immune response [187]. Furthermore, UVB exposure can increase the density of macrophages in the epidermis and enhance the activity of regulatory T cells (Tregs), potentially disrupting the balance between Th1 and Th2 cells and leading to a Th2-skewed immune response. This shift may be associated with an increased risk of allergic reactions and autoimmune diseases [188].

Keratinocytes can produce a variety of pro-inflammatory cytokines and chemokines to regulate local inflammation and immune responses, thereby promoting the recruitment and activation of immune cells. The intensity and timing of the skin immune response are governed by intrinsic circadian rhythms [189]. For instance, during the early morning, UVR exposure in mice triggers apoptotic sunburn, resulting in the highest expression of pro-inflammatory cytokines such as interferon beta (IFN-β), tumor necrosis factor alpha (TNF-α), interleukin-12 (IL-12 p70), and chemokines like C-X-C motif chemokine ligand 10 (CXCL10) and CXCL1. This correlates with a more pronounced erythema response; in contrast, these reactions are at their lowest levels following afternoon UVR exposure [166]. Within epidermal keratinocytes, the chemokine CXCL14 exhibits significant circadian fluctuations, with a pattern that can initiate innate immune responses to fend off external pathogens. In nocturnal mice, CXCL14 expression is higher during the day and lower at night, whereas in primate skin, its expression is lower during the day and higher at night [190].

In mouse models, the expression of interferon regulatory factor 7 (IRF7) is elevated during the day compared to nighttime. Conversely, in *BMAL1*-deficient transgenic mice, IRF7 expression is increased, accompanied by a rise in serum IFN-β levels that lack a clear pattern [191]. This indicates that the core clock gene *BMAL1* acts as a negative regulator of IFN-β release from keratinocytes, with responses being time-dependent and influenced by feeding schedules [191]. In human keratinocytes, TIMP3 influences signaling pathways by inhibiting metalloproteinase activity, subsequently affecting the secretion and activity of cytokines, thus mitigating skin inflammation. UVB radiation decreases TIMP3 expression in human keratinocytes via the *CLOCK* gene, leading to the overexpression of factors such as TNF-α, IL-8, and CXCL1 [182]. TNF-α enhances antigen presentation and induces the release of pro-inflammatory cytokines and chemokines, thereby regulating the recruitment of immune cells. Both IL-8 and CXCL1 are chemokines that promote the chemotaxis and activation of neutrophils, facilitating their arrival at inflammation sites and supporting skin regeneration and repair [192,193,194].

**Figure 2:**
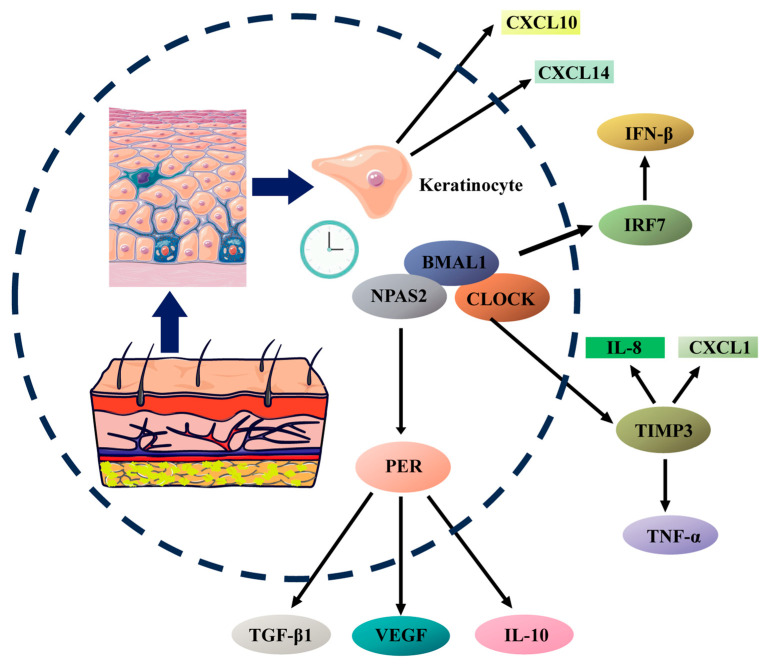
Circadian regulation of cytokine and chemokine production in keratinocytes.

Circadian rhythms can also modulate the expression of transforming growth factor beta 1 (TGF-β1), vascular endothelial growth factor (VEGF), and interleukin-10 (IL-10) in keratinocytes by affecting the activity of transcription factors such as NF-κB and AP-1 [195]. TGF-β1, an important growth factor, is involved in cell proliferation and tissue repair, promoting fibroblast migration and collagen synthesis [196]. VEGF increases vascular permeability and enhances local blood supply, providing more oxygen and nutrients [197]. IL-10 can inhibit excessive inflammatory responses, helping to maintain the balance of the local microenvironment [198]. One of the core clock genes, *NPAS2*, regulates the expression of *PER1* and *PER2* genes, promoting the expression of TGF-β1 and VEGF while inhibiting IL-10 expression, thereby modulating skin functions [195,199]. The regulation of inflammation and immune responses by keratinocytes under circadian rhythms is illustrated in Figure 2.

Beyond the skin, circadian influences on cytokine and chemokine expression have also been observed in other tissue cells. In the hearts of *BMAL1*-deficient mice, several chemokines (CXCL1, CXCR2, CCR2, CCL6) are significantly upregulated, while genes related to lymphocyte proliferation (such as *CCR7, CCR5, CXCL13*) are downregulated, suggesting that BMAL1 intervenes in cytokine expression to regulate immune responses and inflammation progression [66,200]. In epithelial cells of pneumonia models, BMAL1 affects inflammation responses by modulating the chemokine CXCL5 [201,202]. In bone marrow-derived dendritic cells, knocking down *REV-ERBα* or *REV-ERBβ* leads to an increased expression of pro-inflammatory cytokines such as IL-1β, IL-6, and IL-12β [203].

To sum up, circadian rhythms significantly influence the inflammatory and immune responses in the skin, particularly in keratinocytes. These rhythms modulate the secretion of various pro-inflammatory cytokines and chemokines, thereby affecting how the skin responds to UV-induced damage.

### 4.5. Oxidative Stress Management

Circadian rhythms significantly influence oxidative stress in the skin following UVR exposure. UVA radiation can both directly trigger the generation of ROS at the cytoplasmic and membrane levels and indirectly promote ROS accumulation through mitochondrial damage, leading to oxidative stress and subsequent damage to nucleic acids, proteins, and lipids [79]. Excess ROS can produce 8-oxodG (8-oxo-2′-deoxyguanosine), which exacerbates UVR-induced DNA damage and apoptosis, compromising genomic stability and increasing the risk of malignant transformation [204].

In wild-type mice, ROS levels exhibit a clear circadian variation, peaking at 2 p.m. and reaching a nadir at 2 a.m. [52]. In *BMAL1* knockout mice, ROS levels are significantly elevated, and the circadian regulation of oxidative phosphorylation-related genes and ROS levels is lost. This indicates that BMAL1 ensures DNA replication occurs during periods of minimal oxidative stress, indirectly maintaining normal DNA replication processes and protecting cells from DNA damage [52]. Circadian rhythms regulate oxidative phosphorylation in keratinocytes, separating it from the cell proliferation cycle, thereby protecting the genome and preventing DNA damage caused by endogenous ROS [52].

Disruption of circadian rhythms exacerbates oxidative stress and cellular damage following UVR exposure. Normally, circadian rhythms regulate the glycolysis and oxidative phosphorylation processes in proliferating epithelial stem cells, which cyclically transition over time. During daylight hours, cells predominantly rely on oxidative phosphorylation for energy production, a process in which ROS generation is relatively controlled. In contrast, during the dark phase, cells rely more on glycolysis, resulting in lower ROS levels and protecting DNA from oxidative damage, particularly during the DNA-sensitive S phase [205]. Specifically, OGG1 is a key enzyme for removing oxidative DNA damage such as 8-oxoG. In human fibroblasts, knockdown of *BMAL1* not only abolishes the circadian oscillation of OGG1 but also increases its activity, accelerating the repair of oxidative damage [83]. When circadian rhythms are disrupted, the timing of metabolic transitions is misaligned, leading to increased ROS generation during the day and insufficient reduction of ROS at night. This disruption in metabolic rhythm makes cells more susceptible to ROS accumulation under UVR exposure, potentially resulting in oxidative stress, DNA damage, cellular dysfunction, and accelerated skin aging and disease development.

The mammalian target of rapamycin complex 1 (mTORC1) is known for its role in promoting ROS accumulation and inducing replicative senescence [206]. Inhibition of mTOR by rapamycin reduces ROS formation and oxidative stress by upregulating mitochondrial superoxide dismutase (MnSOD) expression. In *BMAL1*-deficient models, both in vivo and in vitro, mTORC1 activity is significantly increased [207]. This suggests that BMAL1-mediated mTORC1 inhibition may play a critical role in reducing oxidative stress and cellular damage following UVR exposure. By regulating mTORC1 activity, BMAL1 influences ROS production and protects cells from UVR-induced oxidative damage.

Circadian rhythm genes also interact with key molecules in cellular signaling pathways, such as MAPK and PI3K/Akt pathways, influencing the expression and activity of antioxidant enzymes. BMAL1 and CLOCK, as core circadian regulators, modulate the activity of 3-phosphoinositide-dependent protein kinase 1 (PDK1) [208]. When PDK1 is activated, it specifically phosphorylates Thr308 of AKT and activates it [209]. Phosphorylated AKT can regulate the transcription and translation of antioxidant enzymes, including superoxide dismutase (SOD) and glutathione peroxidase (GPx), either directly or indirectly [210]. AKT can enhance antioxidant enzyme expression and activity by inhibiting certain negative regulators, such as FOXO transcription factors [211,212,213]. In macrophages, CLOCK and BMAL1 also regulate the accumulation of Nrf2 protein in a circadian manner, triggering the rhythmic expression of its downstream antioxidant target genes and inhibiting the generation of ROS and pro-inflammatory cytokines (e.g., IL-1β) [184,214].

Circadian rhythm genes not only control Nrf2 but also regulate the activity and function of various transcription factors, including FOXO (forkhead box class O) family members, peroxisome proliferator-activated receptors (PPARs), and hypoxia-inducible factor 1-alpha (HIF-1α). FOXO transcription factors are activated in response to oxidative stress and stimulate the transcription of antioxidant enzyme genes by interacting directly with their promoters, enhancing the cell’s resistance to oxidative damage [215]. PPARs can also increase the expression of antioxidant enzymes (e.g., catalase CAT, superoxide dismutase SOD) and effectively reduce intracellular ROS levels, thereby protecting cells from oxidative stress [216]. HIF-1α can directly activate various antioxidant enzyme genes, such as *SOD*, *GPx*, and *CAT*, to clear excess ROS from cells [217,218]. Many antioxidant enzyme genes are potentially regulated by the core circadian rhythm regulators BMAL1–CLOCK complex [219]. Antioxidant enzyme genes, including *SOD1* and *SOD3*, *GPX1* to *GPX4* and *GPX6*, *CAT*, and thioredoxin reductase (*TXNRD1*), are rich in E-box sequences within their promoter regions [220]. The BMAL1–CLOCK complex can specifically bind to these E-box sequences to regulate the transcriptional activity of antioxidant enzyme genes, ensuring that cellular redox homeostasis fluctuates with the circadian rhythm and effectively scavenges ROS, thereby reducing oxidative stress and cellular damage. This network of factors, regulated by circadian rhythm genes, constructs a complex antioxidant defense system [221].

### 4.6. Hormonal Signaling

The skin is widely recognized as a multifunctional neuroendocrine organ capable of producing and responding to various hormones and neurotransmitters [222,223]. Among these, melatonin, a key circadian rhythm regulator, is synthesized in the skin and plays a crucial role in various skin functions, including hair growth, recovery from UVR-induced damage, wound healing, and anti-tumor effects [224,225]. Additionally, the skin produces corticosteroids, which are vital for controlling inflammatory and immune responses.

Melatonin is an indoleamine synthesized by the pineal gland during dark periods and converts light cycle information into hormonal signals [226,227]. Disruptions to circadian rhythms significantly suppress melatonin secretion. For instance, women who have worked night shifts for over 20 years have nearly an 80% higher relative risk of breast cancer compared to those who have never worked such shifts [228,229].

Both physiological and pathological conditions can induce local melatonin production in the skin [230]. Notably, melatonin levels in the human epidermis are higher than those in the blood, indicating its significant physiological role in the skin [231,232]. Acute UVR exposure leads to a notable decrease in melatonin levels due to feedback inhibition [233]. Specifically, UVR activates photoreceptors in the skin, which in turn suppress melatonin secretion from the pineal gland. This feedback mechanism results in peak melatonin levels at night and low levels during the day, especially during intense UVR exposure [233].

Melatonin and its metabolites exhibit strong antioxidant properties, counteracting ROS production and protecting cells from mitochondrial and DNA damage, thus mitigating carcinogenesis and inflammation [234,235,236]. Research indicates that melatonin is more effective than vitamin C or vitamin E in scavenging ROS [237]. After UVR exposure, the nighttime peak of melatonin can enhance the activity of DNA repair enzymes, such as OGG1, accelerating the repair of oxidative damage [238,239]. Melatonin itself has powerful antioxidant and DNA repair capabilities, and its active metabolites, such as AFMK (N1-acetyl-N2-formyl-5-methoxykynuramine) and NAS (N-acetylserotonin), can enhance the expression of antioxidant enzymes like glutathione synthetase (GCS), GSTP1, GPX1, CAT, CuSOD, and MnSOD [240,241]. These metabolites directly neutralize free radicals generated by UVR radiation, reducing DNA damage and preserving cell membrane integrity and function, thereby mitigating inflammation and cellular damage following UVR exposure.

Melatonin influences cellular energy metabolism and adaptation by modulating the activity of nicotinamide phosphoribosyltransferase (NAMPT), which affects NAD^+^ salvage synthesis pathways [242,243]. NAD^+^ is a critical electron carrier in the mitochondrial respiratory chain, essential for the tricarboxylic acid cycle and oxidative phosphorylation, and is a substrate for PARPs (poly(ADP-ribose) polymerases), which play key roles in DNA repair. By maintaining adequate NAD^+^ levels, melatonin indirectly promotes DNA repair and genomic stability [242,243].

UVR can disrupt the skin’s circadian rhythms, and the circadian release of melatonin helps restore normal skin rhythms, promoting repair processes in damaged skin [58]. Exogenous melatonin can regulate circadian rhythms in prostate cancer cells by upregulating the expression of core clock genes such as *PER2* and *CLOCK*, while downregulating the levels of *BMAL1* [244]. On the other hand, melatonin indirectly affects the acetylation state of the BMAL1–CLOCK complex by inhibiting SIRT1, an NAD^+^-dependent histone deacetylase, thereby regulating the normal functioning of circadian rhythms [245].

In addition to melatonin, cortisol, a key hormone involved in peripheral tissue clock synchronization, can indirectly influence the skin’s response to UVR damage by regulating the circadian expression of KLF9 (Krüppel-like factor 9) [148]. Higher cortisol levels in the morning enhance KLF9 expression, inhibiting keratinocyte proliferation and reducing cell sensitivity to UVR damage. Conversely, lower cortisol levels at night weaken KLF9 expression, potentially allowing more active cell proliferation and repair mechanisms, which are beneficial for repairing UVR-induced damage sustained during the day [148].

## 5. Circadian Influence on Skin Wound Healing Processes

As the largest organ of the human body, the skin’s wound healing process is regulated by circadian rhythms. This process encompasses five stages: hemostasis, inflammation, proliferation, re-epithelialization, and remodeling [246]. Studies have shown that hamsters subjected to damaging light exposure exhibit longer wound healing times compared to those maintaining a normal circadian rhythm [247]. Additionally, mice exposed to dim light at night before injury also show significantly delayed wound healing [248]. Mouse models with a knockout of *BMAL1* and *NONO* demonstrate severe wound healing defects, while the partial or complete knockout of *NPAS2* (a CLOCK homolog) significantly shortens the wound healing time in mouse skin [73,249]. Clinical observations indicate that wounds healing during the day typically recover faster than those at night, with nighttime burn healing times in humans extending by approximately 60% [250]. Thus, selecting appropriate timing for surgeries or wound care in clinical practice may enhance healing outcomes.

The early stage of wound healing involves the formation of a clot at the injury site to control bleeding and activate inflammatory cells [251]. The hemostasis process involves platelet aggregation and the activation of coagulation factors [252]. During this phase, plasminogen activator inhibitor-1 (PAI-1) peaks in the morning, coinciding with increased platelet aggregation and coagulation activity [253]. This suggests that the body’s coagulation ability is enhanced in the morning, facilitating rapid wound closure, reducing bleeding risk, and promoting clot formation [254]. Conversely, levels of tissue-type plasminogen activator (t-PA) and D-dimer significantly increase in the afternoon, aiding in the clearance of formed clots and enhancing blood circulation, thereby providing a favorable environment for new cell migration and tissue regeneration. This indicates that circadian rhythms influence the wound healing process by modulating the activity of the fibrinolytic system [253,255,256]. The activation of inflammatory cells occurs swiftly after hemostasis [257]. Under circadian regulation, the elevation of PAI-1 can attract inflammatory cells, such as neutrophils and macrophages, to the injury site, aiding in wound cleansing and the release of cytokines to modulate immune responses and promote healing [253]. Additionally, the S phase of the cell cycle is a crucial stage for cell growth and DNA replication; under circadian regulation, a more active S phase during the night promotes the proliferation of keratinocytes, aiding in skin regeneration and repair, particularly in the early stages of wound healing [67].

In the mid-stage of wound healing, keratinocytes proliferate to fill the surface defect of the wound, facilitating re-epithelialization. Migrating keratinocytes at the wound edge form a new epithelial layer, promoting wound closure [258]. Fibroblasts not only proliferate to produce new extracellular matrix (ECM) for support and structure but also synthesize key components such as collagen and fibronectin, promoting wound healing and enhancing tissue mechanical strength [259]. The core clock genes in these cell types, such as *BMAL1* and *CLOCK*, exhibit significant circadian variation, leading to different activity levels at different times. When skin is injured, clock genes periodically regulate the actin cytoskeleton and cell adhesion mechanisms, resulting in fibroblasts showing higher migratory capacity during the day, thus accelerating the healing process [260]. Furthermore, fibroblasts in the S phase of the cell cycle proliferate rapidly during the day, producing more ECM components like collagen and fibronectin, which are essential for stabilizing and healing the wound in the mid-stage [261]. In keratinocytes, Krüppel-like factor 9 (Klf9), an epidermal transcription factor associated with proliferation inhibition, peaks in expression in the morning. Its inhibitory effect weakens at night, promoting cell proliferation and creating favorable conditions for wound healing [148]. The cortisol hormone induces Klf9 expression, peaking in the morning and gradually declining with the onset of night. However, if circadian rhythms are disrupted and cortisol levels are abnormally elevated at night, this not only inhibits fibroblast proliferation but also affects Klf9 expression, thereby impairing skin repair capacity [148]. Circadian rhythms can also influence the generation and clearance of ROS, affecting cells’ responses to oxidative damage [219]. Fibroblasts and keratinocytes exhibit lower ROS levels at night, enhancing cell viability and functionality, thus promoting wound healing [262].

In the late healing phase, circadian rhythms regulate fibroblast proliferation and maintain the balance of ECM synthesis and degradation, preventing excessive collagen deposition, which contributes to a smoother healing outcome [250].

In summary, circadian rhythms play a critical role in the skin wound healing process. By regulating cell proliferation, migration, gene expression, hormone levels, and oxidative stress, circadian rhythms significantly influence wound repair capacity. Future research may leverage the effects of circadian rhythms to enhance wound healing outcomes, improving patient recovery experiences and quality of life.

## 6. Conclusions

Circadian rhythms, the internal clock mechanisms of organisms, are increasingly recognized for their role in skin health, particularly in the context of UVR exposure and associated skin disorders. This intrinsic rhythm not only influences the skin’s self-renewal and wound healing capabilities but also meticulously regulates various biochemical processes. Circadian rhythms modulate DNA repair mechanisms in skin cells, ensuring that damage is repaired at optimal times, which enhances repair efficiency and reduces mutation accumulation. Additionally, circadian rhythms regulate the expression of cell cycle proteins and mitotic factors, influencing the proliferation rate of skin cells and affecting the skin’s ability to self-renew and recover from UVR damage.

Furthermore, circadian rhythm genes control apoptotic pathways, facilitating the timely removal of damaged or senescent cells and preventing their accumulation and potential carcinogenesis. They also affect the production of inflammatory factors such as interleukins and tumor necrosis factor, thereby controlling the intensity and duration of inflammation and preventing tissue damage from excessive inflammatory responses. Circadian rhythms regulate the activity of antioxidant enzymes, including superoxide dismutase and glutathione peroxidase, to combat free radicals generated by UVR exposure, protecting cells from oxidative stress. Moreover, circadian rhythms influence the secretion of hormones such as cortisol and melatonin, indirectly regulating skin repair processes and immune responses, thus enhancing the skin’s resistance to external challenges.

**Figure 3:**
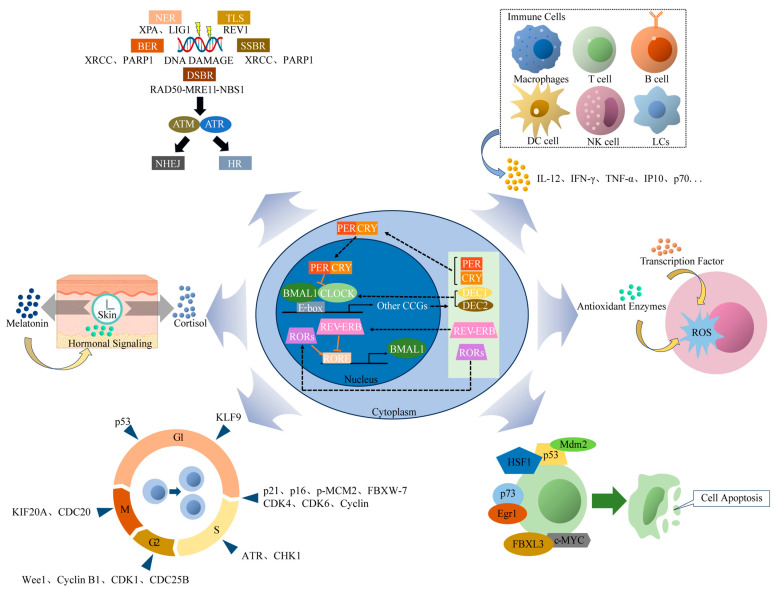
Overview of circadian rhythms in UVR-induced skin damage and repair.

In summary, circadian rhythms constitute a complex defense network that addresses the challenges posed by UVR exposure and other environmental factors (Figure 3). Understanding this network’s mechanisms is crucial for developing more effective skin protection and treatment strategies, including time-specific skincare regimens and chronobiological drugs. Future research will delve deeper into the intricate relationship between circadian rhythms and skin health, aiming to provide new insights for the prevention and treatment of skin diseases.
